# Immune System Acts on Orthodontic Tooth Movement: Cellular and Molecular Mechanisms

**DOI:** 10.1155/2022/9668610

**Published:** 2022-10-25

**Authors:** Yajun Gao, Qingqing Min, Xingjia Li, Linxiang Liu, Yangyang Lv, Wenjie Xu, Xudong Liu, Hua Wang

**Affiliations:** ^1^Department of Endodontics, Wuxi Stomatology Hospital, Wuxi, China; ^2^Department of Prosthodontics, Wuxi Stomatology Hospital, Wuxi, China; ^3^Department of Implantology, Wuxi Stomatology Hospital, Wuxi, China; ^4^Wuhu Stomatology Hospital, Wuhu, China

## Abstract

Orthodontic tooth movement (OTM) is a tissue remodeling process based on orthodontic force loading. Compressed periodontal tissues have a complicated aseptic inflammatory cascade, which are considered the initial factor of alveolar bone remodeling. Since skeletal and immune systems shared a wide variety of molecules, osteoimmunology has been generally accepted as an interdisciplinary field to investigate their interactions. Unsurprisingly, OTM is considered a good mirror of osteoimmunology since it involves immune reaction and bone remolding. In fact, besides bone remodeling, OTM involves cementum resorption, soft tissue remodeling, orthodontic pain, and relapse, all correlated with immune cells and/or immunologically active substance. The aim of this paper is to review the interaction of immune system with orthodontic tooth movement, which helps gain insights into mechanisms of OTM and search novel method to short treatment period and control complications.

## 1. Introduction

Orthodontic tooth movement (OTM) is a complicate remodeling of biological tissue in dentoalveolar complex. Classic “pressure-tension” theory proposes that mechanical force is capable of compressing periodontal ligament (PDL) and reducing blood flow on the compression side [1]. Cytokine secretion and oxygen tension decreasing lead to temporary sterile inflammation [2], followed by a wide variety of immune cells activation. The activated immune cells (e.g., T cell) have been found to play a crucial role in orthodontic treatment [3–5]. The immune–bone interaction has been confirmed in different systemic diseases [6, 7]. Jiang et al. reviewed the effect of immune-related cytokines on orthodontic bone remolding [8], whereas they focused on regulation of osteoclast/osteoblast. In fact, OTM has been reported as a more complicated process than pure bone remodeling. This review places a focus on other aspects in OTM concerned by orthodontists and patients (e.g., cementum resorption, soft tissue remodeling, orthodontic pain, treatment for patients with diabetes, and orthodontic relapse). The multidimensional illustration of role of immune in OTM takes on a critical significance to understand underlying mechanisms and optimize clinical strategies.

## 2. Alveolar Bone Remodeling

PDL is a fibrous structure connecting the cementum on the tooth root surface to the alveolar bone and fix the tooth in the alveolar socket [9], which makes tooth movement possible. PDL is compressible to attenuate the occlusal stresses. When tooth subjects lateral force, it moves slightly along the direction of force, and the actions of PDL are different on the compression side and the tension side. On the compression side, the vessels are squeezed and local ischaemia ensues [10]. Moreover, oxygen tension changing incites an increased anaerobic respiration in periodontal cells (e.g., fibroblasts). Chemical microenvironment alteration contributes to the production of mediators (e.g., prostaglandins), while adjusting the balance between osteoclast and osteoblast to enhance bone remodeling. Osteoclast differentiation is facilitated, thus resulting in alveolar bone resorption on the compression side. In contrast, the PDL fiber is stretched on the tension side, meanwhile, osteoblast activity increases and bone trabecula forms along direction of force applied. Besides, cementum covered tooth root is less susceptible to stress, thus revealing that tooth root will not remodel like alveolar bone, which is the biological basement for orthodontic tooth movement. Ultimately, intact tooth moves in alveolar bone to relieve the compression of PDL.

### 2.1. Osteoclasts

**Figure 1:**
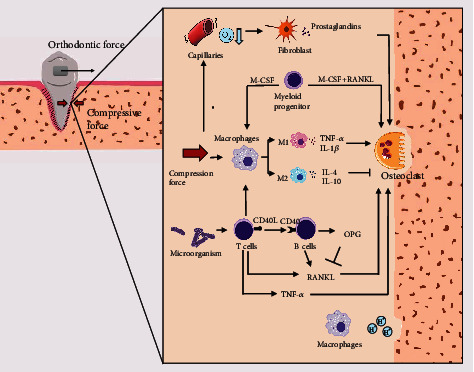
Mechanisms of action in the interaction of osteoclasts with the immune system in orthodontic tooth movement. Compression force squeezed capillaries to reduce oxygen tension, which leads prostaglandins secretion by fibroblast and osteoclastic activation. Myeloid progenitor differentiates to osteoclast in the presence of M-CSF and RANKL, but to macrophage under stimulus of only M-CSF. Macrophages can not only demineralize bone to construct attachment site for osteoclast but also be directly activated by compression force and alter immune microenvironment to regulate osteoclast. T cells are capable of enhancing macrophage M1 polarization and interacting with microorganisms, which promote proinflammatory cytokine secretion and osteoclast activity. Meanwhile, T cells and B cells both express RANKL to facilitate osteoclast differentiation. In addition, T cells express CD40L to bind to CD40 to induce B cells to release OPG which competitively combined to RANKL to inhibit osteoclasts.

Osteoclast is one of the critical bone cell populations.  Figure 1 presents the role and interaction of osteoclasts with immune system in OTM. Osteoclasts and osteal macrophages competitively differentiate from myeloid progenitor. The myeloid progenitor can differentiate into osteoclasts in the presence of macrophage colony-stimulating factor (M-CSF) and receptor activator of NF-*κ*B ligand (RANKL), whereas it turns into mono macrophage under the stimulus of M-CSF alone [11]. Moreover, it has been confirmed that macrophage can affect osteoclast formation and activation [12]. First, activation of macrophage leads to low pH and local bone demineralization to construct attachment site for osteoclast [13]. Second, macrophage is highly heterogeneous, which refers to different functional phenotypes it exhibits in response to local changes in the microenvironment, such as alteration of cytokines, pH-value, and metabolite even oxygen concentrations [14]. For the sake of understanding and discussion, it is generally categorized into M1, proinflammatory, classically activated macrophages associated to initial inflammation, and M2 anti-inflammatory, alternatively activated macrophages which limits inflammation and tissue injury. It is noticeable that macrophage polarization states are much more complex, and M2 macrophages can be further subdivided in M2a, M2b, M2c, and M2d. However, roles of these subtypes in osteoimmunology remain unclear, so below discussion is still based on classic categorization. M1 macrophages secrete TNF-*α* and IL-1*β* to induce osteoclastogenesis, while IL-4 and IL-10 released by M2 macrophages suppress osteoclastogenesis by inhibiting NFATc1 [12], thus revealing that myeloid lineage cells adjust its own differentiation direction by changing external inflammatory environments. On the other hand, the polarization states of macrophage are consecutively and interconvertible, so the influences of macrophages on osteoclast are not fixed.

The interaction between innate immune and osteoclast takes on a critical significance during OTM. To facilitate osteoclast formation, M1/M2 macrophage ratio is increased to exert a more significant proinflammatory effect [15, 16]. The enhancement can be achieved by TH1 cytokines [17, 18] and NLRP3 inflammasome from periodontal ligament cell (PDLC) [19]. Moreover, autophagy of periodontal ligament stem cells (PDLSC), a kind of stem cell with multilineage differentiation potential in periodontal ligament, can promote M1 polarization via inhibiting the AKT signaling pathway as well [20]. Besides, orthodontic mechanical force affects innate immune cell by directly acting on macrophages. The macrophages subjected to compressive force express cytokines rapidly after the application of force (e.g., MMP-8, TNF-*α*, IL-6, PGE-2, and VEGF) [21]. The above cytokines facilitate immune cell recruitment, vessel growth to aggregate inflammation, thus accelerating osteoclast differentiation and tooth movement [22–25]. Notably, oxygen plays a certain role in this regulation of macrophages since TNF is inhibited partly by increased oxygen [26]. Thus, hypoxia-associated protein (e.g., hypoxia-inducible factor-1 (HIF-1)) can be the target for modulating macrophages to optimize orthodontic treatment. Complement is another essential part of innate immunity, which has been considered to affect bone homeostasis [27]. Ignatius et al. suggested that osteoblasts and osteoclasts are capable of activating complement by cleaving complement component 5 (C5) to its active form C5a. Furthermore, C3a and C5a induce osteoclast formation even in the absence of RANKL and M-CSF [28]. The above findings reveal that complement is capable of modulating osteoclast formation independently.

RANKL/RANK/OPG system has been confirmed as the critical pathway for osteoblast to regulate osteoclast. RANKL, one of the TNF family members, binds to RANK, a receptor expresses on osteoclastic progenitor cells, to mature osteoclast [29]. By binding to RANKL and blocking it, OPG is a decoy receptor that plays a certain role in the prevention of extra bone resorption as a bone protective factor [30]. Skeletal system achieves bone homeostasis by maintaining dynamic balance between bone resorption and deposition via RANKL/RANK/OPG pathway. In orthodontic treatment, it has been found that orthodontic force increases the concentration of RANKL in gingival crevicular fluid, thus activating osteoclasts and increasing the amount of experimental tooth movement in rats [31, 32]. In contrast, the overexpression of OPG in rat periodontium inhibits bone remodeling and OTM [33]. However, RANKL/RANK/OPG system is easy to intervene because they are expressed not only in osteoblast. Entire B cell lineage including B cell precursors, immature B cells, and plasma cells account for about 64% of total OPG production. Meanwhile, B cell also expresses RANKL that leads to bone loss. Both increase as aging but OPG cannot fully compensate for endogenous RANKL for the elders [34, 35]. When RANKL gene is deleted from B cells in mice, bone loss arising from ovariectomy is partially reversed [36]. The above research has indicated that B cell can be a potential target to reverse bone resorption resulted from age-associated OPG-RANKL imbalance. Moreover, systemic immune system diseases affect bone metabolism by modulating B cells. The flow cytometry results have suggested that HIV-infected individuals received B cell dysmaturity, thus contributing to decreased bone mass and OPG, as well as osteoporosis [37]. Meanwhile, B cell is considered a source of RANKL to facilitate osteoclastogenesis in periodontitis. The above evidence suggests that B cells can activate osteoclasts by regulating RANKL and OPG levels, and they influence periodontium. However, the direct evidence supporting that B cells directly modulate bone remodeling induced by orthodontic force is scanty. The similar situation pertains to the T cell, another type of important immune cell capable of regulating bone homeostasis. T cells induce B cells by expressing CD40 ligand which binds to CD40 on the B cell surface to release OPG [38]. Li et al. [38] observed that T-cell-deficient nude mice express lower level of B cell-derived OPG. Interestingly, activated T cells can initiate bone destruction by releasing TNF-*α* in inflammation and under pathological conditions (e.g., estrogen deficiency) [39]. It is also capable of secreting IFN-*γ* which polarize macrophages into M1 [40, 41] and releasing RANKL which induces osteoclasts differentiation by binding to RANK [42, 43]. Furthermore, inhibition of T cell-derived RANKL by microRNA-21 leads to OTM retard in mice [4], thus revealing that the T cell affects osteoclast activity in orthodontic treatment, which can explain why T cell is required for OTM [3].

Besides directly regulating cells, immune system also interacts with foreign microorganisms to affect bone homeostasis. Recent reports showed that probiotics have impacts on immune system and bone [44–47]. It is widely accepted that probiotics can maintain bones by reducing inflammatory factors. In particular, Sjögren et al. found that CD4^+^ T cells along with TNF-*α* and IL-6 they secreted decline in bone marrow, serum, and spleen in Germ-free mice, thus indicating the decrease of osteoclastogenesis and the increase of bone mass in absence of intestinal flora [48]. Despite oral cavity is a part of digestive system harboring the second most abundant microbiota after the gastrointestinal tract [49], the interaction between probiotics and oral flora is still ambiguous compared to intestinal flora. On the other hand, some study have reported that oral supplementation of *Bacillus subtilis* reduces alveolar bone loss in rats with periodontitis [50] and decreases alveolar remodeling in OTM [51], even though underlying mechanism remains unknown. Anyway, more investigations are necessary, and it is expected that probiotic therapy can be clinically used in periodontal or orthodontic treatment in the future.

Hormone has been widely known as an essential regulator of immune system, some of which significantly affect osteoclasts. For instance, adrenal glucocorticoid (GC) is a common inhibitor of immune system since it can suppress cytokine secretion and induce immune cells death [52]. *In vivo* experiment results have suggested that GC restrains osteoclast activity via GC receptor [53]. Prostaglandin E_2_ (PGE_2_) is another widely known homeostatic factor dominating in late/chronic stages of immunity [54]. PGE_2_ can be synthesized by osteoblastic cell lineage and stimulate osteoclast formation and differentiation [55, 56]. However, some reports about hormones effect have been contradictory. Kaji et al. suggested that dexamethasone enhances osteoclast-like cell formation [57], and Take et al. observed inhibition of human osteoclasts by PGE_2_*in vitro* [58]. The above findings were achieved probably because hormone target cells are so wide range that some of them modulate osteoclast via other pathways. Another important bone homeostasis-associated hormone is estrogen. Postmenopausal women with deficient serum levels of estrogen face a higher risk of osteoporosis [59]. Moreover, ovariectomy-induced mice are considered the most common osteoporosis animal model. Since estrogen significantly prevents osteoclast formation and differentiation while increasing apoptosis by binding to receptors [60], recent research has suggested that mitochondrial deacetylase sirtuin-3 (Sirt3) plays a certain role in the estrogen deficient-induced bone resorption [61]. In addition, a considerable number of types of immune cells express estrogen receptors (e.g., TH1, TH2, mast cell, basophilic cell, B cell, dendritic cell, macrophage, and native CD4^+^ T cell) [62]. Uehara et al. suggested that immune cells play a role of mediator to bridge estrogen and osteocytes and reviewed possible pathways [63]. Parathyroid hormone (PTH) is an 84-amino-acid peptide hormone with great application potential in bone healing. PTH increases the RANKL/OPG ratio to exert osteoclast indirectly [64]. In the bone, PTH increases TNF production [65] and activates T cells expressing CD40L directly [66], positively correlated with osteoclast activity. However, it also has proosteogenic activity since intermittent treatment with PTH analogs is approved as bone anabolic osteoporosis treatment strategy. Anyway, the above evidence emerges that there is interaction of immune cells and osteoclasts.

**Figure 2:**
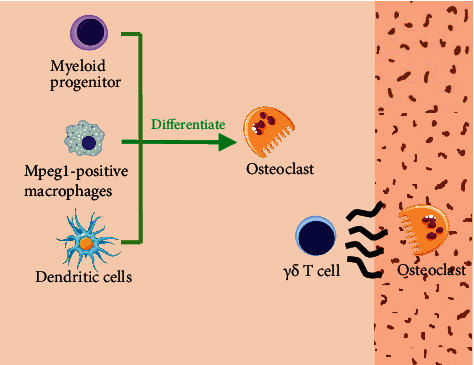
Sources of osteoclasts in alveolar bone. Osteoclast can be derived from myeloid progenitor, mpeg1-positive macrophages, and dendritic cells. Meanwhile, it can be recruited by *γδ*T cell as well.

The major origin of osteoclasts responsible for bone resorption during orthodontic treatment has been found as preosteoclasts recruited from bone marrow [67, 68]. Interestingly, recent studies have reported that some mature immune cells of monocyte lineage can also differentiate into osteoclast under specific stimulation. Dendritic cells (DCs), the most potent antigen-presenting cells, have been found to be transdifferentiated into active osteoclast in the presence of RANKL and M-CSF [69]. Even mpeg1-positive macrophages can be recruited by Cxcl9l from osteoblast progenitors to bone matrix and differentiate into osteoclasts [70]. Moreover, recruitment of osteoclast is correlated with numerous immune cells and cytokines. T cell secretes IFN-*γ*, RANKL, and IL-17A to induce inflammation-promoting osteoclast commitment and bone loss [34]. Furthermore, Wald et al. confirmed that osteoclasts, monocytes, and neutrophils are recruited by *γδ*T cells in mice during OTM [71] (Figure 2).

### 2.2. Osteoblast

Osteoblasts refer to bone-building cells derived from the mesenchymal/mesodermal lineage, which have been identified in tension side of tooth in OTM [72]. During OTM, Cbfa1 and Runx2 have been found as the earliest two transcriptional factors initiating bone formation [29]. In addition, other proteins have been considered as osteogenesis markers, including alpha-smooth muscle actin (*α*-SMA), osteopontin (OPN), osteocalcin (OC), bone morphogenetic proteins (BMPs), transforming growth factor-beta (TGF-*β*), Indian hedgehog, and ostrix [29, 73]. Since the term “osteoimmune” was coined firstly in a publication which described regulation of osteoclastogenesis by T cells [74], the interaction between osteoblast and immune system gradually emerges. The osteoblast-osteoclast coupling is widely accepted, and RANKL/RANK/OPG system referred in the last sections is the most important coupling pathway between them. Moreover, osteoclasts can enhance osteoblast differentiation by expressing membrane-bound ephrinB2 that binds the EphB4 receptor on osteoblast precursors [75], but osteoblast activation precedes commitment of osteoclasts [73]. As osteoclasts resorb bone matrix, factors from the matrix are released to regulate osteoblast activity, which consist of TGF-*β* and insulin-like growth factors (IGFs) [76]. It was reported that some coupling mechanisms between osteoblasts and osteoclasts are involved in immunoregulation. For instance, osteoblast lineage releases M-CSF [77], vascular endothelial growth (VEGF) [78], and nitric oxide [79] necessary for programming of osteoclast formation, which all can interfere immune microenvironment.

In addition, osteoblast directly initiates inflammation under certain conditions. IL-1*β*, C3a, and C5a promote the release of IL-6 and IL-8 in osteoblast [28]. LPS induces osteoblasts to express proinflammatory cytokines through mitogen-activated protein kinase (MAPK) and nuclear factor-*κ*B (NF-*κ*B) signaling pathway [80]. Besides, osteoblasts are the responsive cells for proinflammatory cytokines (e.g., IL-1, IL-11, oncostatin-M, leukemia inhibitory factor, and TNF-*α*). However, numerous studies have suggested that inflammation has a negative effect on bone forming. For instance, IL-1*β* inhibits human osteoblast migration and differentiation [81]. TNF-*α* downregulates Runx2 expression and induces apoptosis in osteoblasts [76]. The above findings have revealed that osteoblast contributes to local inflammation, thus inhibiting osteogenesis from the formation of a self-negative feedback regulatory mechanism. However, effect of immune response is not always negative for osteogenesis. It is reported that osteoclast-derived C3a induces osteoblast differentiation [82], although Ignatius et al. had negative results in another similar *in vitro* research employing C3a and C5a [28]. Thus, further investigation is needed.

B cells also interact with osteoblast. B cell is a key regulator of fracture healing and inhibits excessive bone regeneration by producing multiple osteoblast inhibitors [83]. Osteolineage cells are considered to play a role in the establishment of the niche where B lymphopoiesis occurs, and abnormal osteoblastic function may result in the defect of B lymphopoiesis [84]. Some evidence has suggested that calvarial osteoblasts can support B-cell commitment and differentiation from hematopoietic stem cells (HSCs) [85]. It is noticeable that B cell infiltration is observed in alveolar bone loss induced by periodontitis, which is correlated with activation of RANKL pathway as well as low levels of memory B cells [86, 87]. However, the effect of interaction between B cells and osteoblasts on tooth movement is still illegible. In-depth research should be conducted.

Like osteoclast, osteoblast is also the target of immune-associated hormones. Glucocorticoids inhibit osteoblastogenesis and promote apoptosis of osteoblasts, thus causing osteoporosis during its long-term use [88]. Parathyroid hormone acts directly on osteoblast and stimulates its differentiation and formation via STAT3/*β*-catenin during OTM [89–91]. Li et al. also observed upregulated osteogenic proteins OC, ALP, and IGF-1 in the gingival cervical fluid of mice after intermittent parathyroid hormone treatment [92]. There is substantial evidence to support the effect of estrogen deficient on bone mass and bone microarchitecture [93–95]. This is because estrogen deficient destroys directly bone forming induced by osteoblast, though this effect is milder than activation of osteoclast. Another possible reason can be increased inflammatory cytokines levels in the postmenopausal patients (e.g., TNF*α*, IL-1*β*, and IL-6), which have impact on bone homeostasis [93]. In brief, the interaction between hormone and osteocyte should be considered during orthodontic treatment of patients with special hormonal status.

## 3. External Root Resorption

Alveolar bone remodels under compression while tooth root is protected by cementum from the force [96]. Discrepancy in their response is considered the biological basis of orthodontic tooth movement. Nevertheless, destruction of root, termed the external root resorption (ERR), can still occur on mineralized cementum or dentine due to the presence of osteoclast-like multi, or occasionally mononucleated cells, called odontoclasts [97]. External root resorption occurring during orthodontic treatment has been confirmed as an iatrogenic disorder due to sterile inflammation [98, 99]. Histological root resorption is detected in over 90% orthodontically treated teeth, and the incidence of root resorption below 2 mm ranges from 48% to 66% [100, 101]. This noninfective, slight resorption is known as external surface resorption (ESR) which can only be confirmed with radiographs. It stops once orthodontic pressure is removed. Teeth subjected ESR suggest blunted root apices and/or the shorter roots. Moreover, due to the repairment of cementum, this resorption is also considered self-limiting and localized [97, 102]. However, 5% orthodontic patients' ERR can increase to more than 5 mm [103], which is the reaction to excessive orthodontic mechanical stimulus. Besides, the detailed mechanism remains unclear.

Osteoclasts and odontoclasts responsible for root resorption are significantly similar to each other. There are some parallels in their response to immune regulation. He et al. [16] suggested the induction of ERR by proinflammatory macrophage phenotype. Increased M1/M2 ratio upregulates the expressions of TNF-*α*, IFN-*γ*, and nitric oxide, while anti-inflammatory cytokines, IL-10, and arginase I are inhibited in OTM. Enhanced inflammation leads to assemblage of odontoclasts/osteoblast and external root resorption in rats, which reveals that some immune cells that modulate macrophage polarization (e.g., helper T cells [104]) can have an effect on root resorption. NLRP3 inflammasome also positively regulates M1 macrophage to exaggerate root resorption through caspase-1/IL-1*β* signaling [19]. Furthermore, the biological reaction in OTM can involve systemic cellular recruitment since spleen reservoir monocytes are significantly reduced [105]. The regional inflammation and root resorption are attenuated by a systemic level of TNF-*α* inhibitor etanercept or IL-4 [16]. The above evidence suggests the correlation between regional complication of orthodontic and systemic immune system.

However, in contrast to conventional knowledge that inflammation always exacerbates ERR, the risk of root resorption is confirmed to be negatively correlated with classical proinflammatory cytokine IL-1*β* which stimulates osteoclast recruitment and alveolar resorption. P2X purinergic receptor 7 is a ligand-gated ion channel which binds to ATP and induce maturation and externalization of downstream IL-1*β* [106–108]. *P2X7* knockout mice were found with more root resorption than wild-type under orthodontic force [109]. Al-Qawasmi et al. knocked out IL-1*β* in C57BL/6J mice and observed root resorption increased [110]. Besides, polymorphism at IL-1*β* genes (+3954) is correlated with production alteration of IL-1*β*, and people with lower IL-1*β* production genotype have higher risks to experience ERR [111]. The above studies support that IL-1*β* is negatively correlated with ERR and explains further why ERR shows clear genetic predisposition. Unfortunately, these reports failed to illuminate why deficient inflammation results in more severe ERR. Prevail theory suggests that deficiency of IL-1*β* inhibits alveolar remodeling, thus resulting in prolonged stress concentrated on the root of the tooth and the destruction of cementum [112, 113]. In other words, the critical initiator of ERR is mechanical stimulation, instead of inflammation. This theory is supported by some reports [114, 115] which observed decreased root resorption rate in animal model of bone loss. It is considered that lower bone density reduces stress in the PDL interface, thus decreasing ERR risk. However, there is controversy about linkage between bone density and root ERR. Though cone-beam CT imaging analysis suggests that lingual bone density is positively correlated with ERR, some clinical trials with a higher sample number achieved negative results [116, 117]. Sirisoontorn [118] even reported that osteoporosis aggravated ERR in mice. Thus, it is necessary to conduct additional studies to further investigate involved mechanisms and explain the above contradictory conclusions.

Apart from IL-1*β* genes, other genetic variants that have been confirmed to be correlated with ERR predisposition include IRAK1, IL-17, IL-6, human vitamin D receptor (hVDR), and OPN genes. Many of them are also correlated with immune response [119–124]. In addition, ERR is also correlated with epigenetic machinery of some immune response-related genes [125]. Existing research has demonstrated that FOXP3 promoters in teeth experiencing ERR have higher levels of DNA methylation, which inhibits transcription factors from binding to their DNA binding sites, thus downregulate the expression of FOXP3. The suppression of FOXP3 genes has an impact on development of ?A3B2 show $132#?>regulatory T cells (Tregs) [126, 127]. It is responsible for maintaining immune homeostasis by antagonizing effector T cells [128]. As more and more studies investigated the association between gene polymorphisms and the risk of EARR during orthodontic treatment, early diagnosis of risk patients is expected in the future.

Besides, some patients with special hormone status can face higher ESRR risk. Cementoblastic activities and the amount of new cementum formation are lower in ovariectomized rats, thus affecting the activation of extracellular signal-regulated kinase-1/2 pathway. Given the similarity between cementoblasts and osteoblasts, it is unsurprising that some scholars manage to reduce EARR by locally injecting a variety of bone forming associated hormones in animals (e.g., estrogen, thyroxine, prostaglandin E2, and parathyroid hormone) [129–132]. However, the underlying mechanisms and the effects of local application of hormones on immune status remain unclear, and it is necessary to conduct further investigations.

## 4. Periodontal Response to Orthodontic Force Application

Existing research has suggested that periodontal soft tissue changes morphologically and functionally in orthodontic treatment [133]. Abnormalities of soft tissue which can be observed in OTM include gingivitis, gingival enlargement, gingival recession, and gingival invagination [134]. Adolescents tend to have higher chances of gingivitis and gingival enlargement compared with adults due to their unstable gonadal hormone level. Gingivitis, the most common soft tissue problem, has been considered to arise from the placement of full-mouth appliance which makes daily oral hygiene care routine more challenging. In fact, the correlation of orthodontic treatment and soft tissue is significantly more sophisticated. The increased retention of particles and dental plaque leads to significant changes of oral flora and salivary pH [135], thus contributing to periodontal diseases and carious lesions development [136, 137]. Naranjo et al.'s research reported increase in periodontal pathogen colonization, scores for bleeding on probing, plaque index, and gingival index after orthodontic bracket placement [138]. Moreover, elevations of the red complex species, *T. forsythia*, *P. gingivalis*, and *T. denticola* in the subgingival biofilm [139], saliva, and the surfaces of brackets [140] have been detected at the early stage or in the middle-term of orthodontic treatment. Though the mean total counts of red complex fall back to baseline level or even lower 12 months after appliance placement [139]. Moreover, subgingival microflora disorder will result in host immune reaction changes. The gingival crevicular fluid (GCF) is the sample employed most frequently since it is easy to collect. GCF from patients with fixed appliance has been reported with a lower anti-inflammatory cytokine (IL-4) level and higher levels of proinflammatory cytokines (TNF-*α*, IL-1*β*, IL-8, and IL-6). IL-6 takes on a great significance in the prediction of GCF flow [137], and TNF-*α*, IL-1*β*, and IL-8 have positive correlation with bleeding on probing and probing depth [137, 141]. However, IL-4 is correlated with nonbleeding sites and no gingival overgrowth [141]. Besides, orthodontic appliance design is capable of modulating dynamic alteration in the oral microbial equilibrium. Removable appliance lowers plaque index and periodontal complications compared with conventional fixed appliance [142, 143]. Bergamo et al. [140] have demonstrated that the structure and material of bracket applied in orthodontic could also affect the level and adherence of bacterial species on it, although exact mechanisms remain ambiguous. Nevertheless, given the correlation of oral microorganisms with periodontal health status, cardiac problems, and immunosuppression [144–147], additional investigations are required to establish effective protocols that prevent periodontal disease, even systemic diseases, by improving orthodontic appliances.

**Figure 3:**
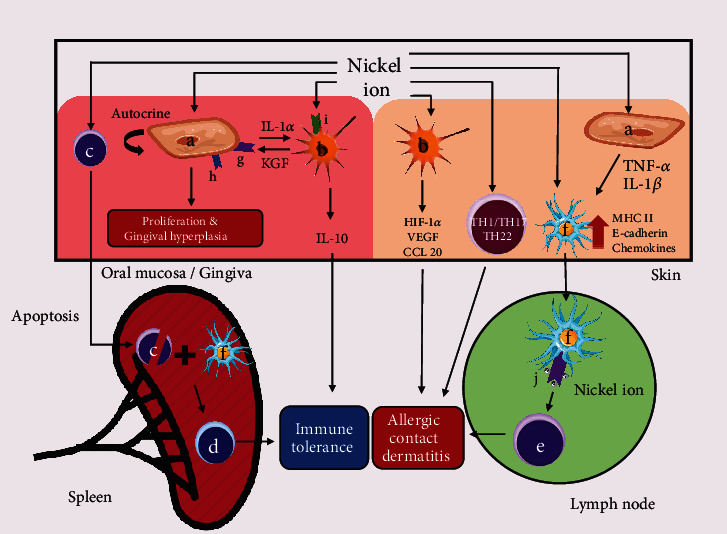
The different effect of nickel ions on oral mucosa/gingiva and skin. (a) Keratinocyte. (b) Fibroblast. (c) B cell. (d) Treg. (e) T cell. (f) APC. (g) KGF receptor. (h) ICAM-1. (i) TLR4. (j) MHC II. In oral mucosa/gingiva, intercellular adhesion molecule 1 (ICAM1) on keratinocyte is elevated by nickel, which facilitates lymphocyte adhesion and chronic inflammation. Nickel ions also increase keratinocyte's proliferation by autocrine pathway to cause gingival hyperplasia. An IL-1*α*/KGF/KGF receptor positive feedback loop is formed between keratinocyte and fibroblast to further stimulate proliferation. On the other hand, fibroblast is irritated to express anti-inflammatory cytokines IL-10 via TLR4, which dampens immune response. Another pathway of forming immune tolerance is dependent on B cell apoptosis, and nickel ions cause DNA strand breaks of B cell to induce splenic apoptosis. APCs capture the apoptotic bodies and cross-present their peptides to the regulatory T cells which is vital immune cells for systemic tolerance. In skin, keratinocyte suffering nickel exposure secretes higher levels of IL-1*β* and TNF-*α* which upregulate MHC II, E-cadherin, and chemokines in APCs. Those APCs present nickel by MHC II molecule to naïve T cells in lymph node to induce allergic contact dermatitis. Another two pathways of dermatitis include that nickel stimulates fibroblast to express HIF-1*α*, VEGF, and CCL20, and that nickel activates elTh1/Th17 and Th22 cells.

Gingival enlargement is another complication that has been commonly identified in juvenile orthodontics patients with unstable gonadal hormone concentrations [148]. In general, it occurs 1-2 months after treatment [149]. In contrast to inflammatory enlargement showing deep red, soft, and easily bleeding lesions, orthodontic treatment-induced gingival overgrowth is thick, pink, and rarely bleeding [150]. Şurlin et al. [151] found overgrowth upon hyperplasia of the basal lamina and hypertrophy of the intermediate layer, accompanied by less foamy acidophilic cytoplasm cells with the absence of chronic inflammatory infiltrate in the chorion. Mechanical irritation by orthodontic bands, chemical irritation by cements, food impaction, and less efficient oral hygiene maintenance has been confirmed as all etiologic factors for gingival enlargement [152]. However, some patients with good oral hygiene also undergo gingival overgrowth. In the above patients' gingival sulcus, an increase in matrix metalloproteinases- (MMP-) 8 level is persisted [153], a collagenase involved in systemic inflammation, orthodontic pain, periodontitis, and persistent virus infection [153–157]. Nevertheless, there have been rare studies revealing the reasons that why orthodontic gingival enlargement patients are more sensitive to external stimulus compared with normal patients. Furthermore, as reported by some existing studies, nickel released from fixed appliance significantly affects gingival hyperplasia (Figure 3). Exposure of skin to nickel can cause allergic contact dermatitis (ACD) in susceptible subjects. In this delayed-type hypersensitivity (DTH) reaction, nickel penetrates the skin tissue and stimulates keratinocytes to secrete IL-1*β* and TNF-*α* which upregulate the major histocompatibility complex II (MHC II) molecule on antigen presenting cells (APCs) in skin, including Langerhans cells (LCs) and DCs. Besides, the above cytokines also regulate E-cadherin and chemokine secretion from APCs. Subsequently, nickel binds to MHC II molecule and is transferred with APCs to draining lymph node where naïve T cells accept happens [158–161]. Nickel would mostly result in immune activation of the Th1/Th17 and Th22 components [162]. In the nickel ion-induced gingival enlargement, keratinocytes are the most important targeted cells. Constant low-dose nickel induces autocrine activation of the keratinocytes to trigger proliferation [163], while increasing intercellular adhesion molecule 1 (ICAM1) expression on surface to facilitate lymphocyte adhesion [164, 165]. Activated keratinocytes release IL-1*α* to stimulate fibroblasts to secrete keratinocyte growth factors (KGF) binding to KGF receptors on keratinocytes to induce proliferation, and the receptor is also upregulated by nickel exposure [166]. Marchese et al. reported that when primary keratinocytes were cocultured with fibroblasts, their proliferative effects would be significantly enhanced [163]. As reported by Gursoy et al.'s research, no significant difference was found in nickel accumulation between samples with or without gingival overgrowth [150]. Thus, etiology of orthodontic-induced gingival enlargement can arise from MHC gene polymorphisms instead of nickel accumulation and nickel content of the fixed appliance. However, some researchers proposed a hypothesis that nickel can accumulate in epithelium rather than connective tissue to create a local high-dose environment. Yet, the above accumulation of nickel in epithelium has been only identified in three-dimensional *in vitro* model [167] and lacks support of mechanistic studies. Another interesting theory is that nickel exposed via oral route can induce tolerance in nickel allergy as immune reaction of human gingival fibroblasts (HGF) is inconsistent with dermal fibroblasts *in vitro* [120]. In comparison with dermal fibroblasts, HGF suffering nickel exposure expresses lower levels of HIF-1*α*, vascular endothelial growth factor (VEGF), and chemotactic cytokines ligand 20 (CCL20). Besides, HGF activated by nickel via TLR4 expresses IL-10, instead of initiating an acute proinflammatory response as in dermal fibroblasts [168]. Another pathway of forming immune tolerance is dependent on B cell apoptosis [169]. Nickel ions cause DNA strand breaks to induce splenic B cell apoptosis, thus activating DNA-damage sensor and the transcription factor p53 [170]. The p53 activation upregulates Fas expression while it downregulates expression of Bcl-xL to promote the apoptosis of B cells in spleen [169]. Furthermore, B cells interact in a CD1d-dependent manner with iNKT cells which express IL-10 and induce B cell apoptosis. In spleen, the above apoptotic B cells are critical to nickel tolerance. Their apoptotic bodies are captured by APCs that cross-present their peptides on MHC-II and MHC-I molecules. As a result, it induces nickel specific CD4^+^ CD25^+^ regulatory T cells which is vital immune cells in terms of systemic tolerance [169, 171–174].

Furthermore, Invisalign aligner is a widely removable plastic orthodontic appliance. Since its constitution does not contain metal, Invisalign system is considered more biocompatible than traditional appliance. However, recent research has suggested that Invisalign aligners change cells behavior and regulate proinflammatory protein expression. This conclusion is consistent with the finding of Premaraj et al. [175]. Invisalign plastic eluent significantly decreases oral epithelial cell viability and barrier function. Interestingly, it also suggested that saliva can protect keratinocyte from eluate. However, the predominate cytotoxic ingredient of Invisalign remains ambiguous. Isocyanate, a component of Invisalign plastic, results in mucous membrane irritation, asthmatic, hypersensitivity reactions, and other health issues. When isocyanate contacts with oral tissues, it rapidly forms immunogenic hapten by binding to protein, thus leading to sensitization. It also leads to oral epithelial barrier disruption which magnifies the allergic reactions [176, 177]. Isocyanate induces an inflammatory reaction and nonspecific airway hyperresponsiveness, involving type 1 and type 2 immune responses [178]. It is observed that isocyanate-exposed animals have greater amounts of airway goblet cells, neutrophils, BAL eosinophils, CD4+ T-cells, and ILCs, with a predominant type 2 response [179, 180]. Thus, workplace exposure of isocyanate is associated with occupational asthma [181]. Besides, 3D resin must be used during the manufacturing process of aligner. The problem is that this 3D resin made of polymethyl methacrylate (PMMA) leaches out residual unpolymerized monomers with toxic effect [182]. Bisphenol A (BPA) is one of the monomers, which is widely known for its cytotoxicity and disruptive effect on endocrine [183]. It can also be detected in orthodontic adhesive [184]. However, it is controversial whether the amount of BPA in the saliva of orthodontic patients is sufficient to affect oral epithelial cells [184–186] since Invisalign plastic does not have the necessary ingredients to release bisphenol. Eliades et al. prepared eluent by immersing aligner for 2 months, and no estrogenic effects were detected [187]. In addition, some supplements may affect cells validity. Rogers et al. [188] reported light stabilizer Tinuvin 292 from dental resin for 3D printing released ovo-toxic leachates *in vitro*. In brief, it is suggested that although some orthodontic biomaterials may have adverse biological effects *in vitro*, there has not been any clinical trial offering solid evidence for cytotoxic of Invisalign aligner in patients' oral environment.

## 5. Orthodontic Pain

Orthodontic pain is initiated by sterile inflammation following orthodontic force, involving vascular changes, cytokines release, and immune cells recruitment [189]. The pain arising from OTM lasts two to three days after appliance placement and tends to decrease by the fifth or sixth day [190]. The duration is dependent on inflammation development and endogenous analgesic mechanisms. What is more, innate immune system can play a part in modulating orthodontic pain as neuroimmune interactions in pain have been confirmed by a plenty of studies [30, 191–193]. Mast cells, neutrophils, macrophages, and T cells are capable of secreting mediators binding to receptors on peripheral nerve terminals to reduce threshold for nociceptor neurons to fire action potentials, i.e., sensitization [191]. In contrast, nociceptors release neuropeptides and neurotransmitters to modulate immune function by influencing immune cells. To be specific, histamine from mast cells stimulates peripheral terminals of nerves in a paracrine manner. Nociceptor neurons express several histamine receptors (including H1R, H2R, H3R, and H4R) correlated with pain sensitization [194]. Upon releasing, tryptase stored in secretory granules of mast cells can interact with protease activated receptor 2 (PAR-2) on nerve endings [195, 196]. The above process induces activation of the transient receptor potential cation channel subfamily V member 1 (TRPV1) channels to upregulate calcitonin gene related peptide (CGRP) and substance P (SP) from nerve terminals [197], thus initiating nociceptive transmission. Other cytokines involved in mater cell-nociceptor interaction consist of IL-5, 5-hydroxytryptamine (5-HT) and nerve growth factor (NGF) [198]. Activated nerve endings release neuropeptides to stimulate a vicious cycle of mast cells further amplifying vascular leakage [194]. Neutrophils and macrophages secrete leukotriene B4 (LTB4), TNF-*β*, IL-6, IL-1*β*, and prostaglandin to activate their receptors and downstream ion channels [191], so as to sensitize nociceptor neurons. Neuroimmune interaction is not limited to peripheral nerve ending. In the spinal cord dorsal horn, T cells and microglia, resident innate immune cell in the spinal cord and the brain, also interact with spinal cord to act on central neuronal sensitization, thus resulting in pain chronicity [199–201].

In OTM, compressed vessels contribute to local ischaemia, thus resulting in anaerobic respiration and local acidosis. The accumulated H^+^ binds to acid-sensing ion channel 3 (ASIC3) on periodontal nerve terminal to induce orthodontic pain [202, 203]. Since orthodontic pain sensation is transferred along axon to trigeminal ganglia, action potentials stimulate periodontal neuron endings for the release of CGRP and SP [189, 204–206]. The above neurogenic mediators upregulate prostaglandins that can modify orthodontic pain [207]. Since orthodontic pain is significantly common in treatment, pain management becomes a major concern for patients and clinicians. Application of NSAID inhibits the production of prostaglandin by reducing the activity of COX enzymes. As a result, orthodontic pain can be alleviated [208]. Opioids are capable of suppressing orthodontic pain by binding to midbrain periaqueductal gray (PAG), so it has been considered a vital site in ascending pain transmission and a major component of the descending pain inhibitory system [209–211]. However, the long-term application of opioids induces significant neuroinflammation since it can activate astrocytes and microglia by Toll-like receptor 4 (TLR4) in the central nervous system [212]. Morphine increases level of glial-derived proinflammatory cytokines, inhibiting expressions of GABA receptors and glutamate transporter proteins. As a result, outward potassium currents decrease, thus leading to an overall increase in excitability of nearby neurons [209, 212–214]. The neuroinflammation results in the reverse of morphine analgesia and the development of morphine tolerance [215], which can explain the reason why administration of lipopolysaccharide (LPS), a potent TLR4 agonist, decreases the analgesic efficacy of morphine [216]. Interestingly, electronic stimulation of dorsal PAG leads to decreased activity of peripheral blood natural killer (NK) cells [217]. Moreover, microinjection of morphine into the caudal aspect of the PAG brings about NK cell cytotoxicity to induce immune inhibition [218]. The mentioned findings reveal the immunoregulatory property of opioids and the role PAG plays.

However, orthodontic pain has been generally self-limited since acute inflammatory reaction is attenuated and tends to be chronic over time [219], and intrinsic analgesic mechanisms have also been found to play a role [220–222]. At the early stage of OTM, nerve fibers in PDL release neuropeptides which elicit a painful response [223, 224]. Nerve fibers containing neuropeptides in PDL of the rat first molar have been found to increase three days after force application and returned to normal after 14 days [225]. Furthermore, PAG has been reported as a critical neural circuit for endogenous opioid-mediated analgesia. The upregulation of endogenous opioids and opioid receptors was identified on trigeminal nucleus during the orthodontic pain response [189, 226]. Orthodontic pain activates PAG to send analgesic signals only at late stages of each episode of orthodontic treatment [227, 228]. Considering the effect arising from pain on the patient compliance and risks for opioids abuse, the mechanisms of pain self-limitation should be clarified systematically, and novel analgesia methods should be explored to replace conventional pain relievers. Guo et al. [229] reported that systemic infusion of bone marrow stromal cells (BMSCs) can interact with monocytes/macrophages by producing chemokines CCL4 and CCR2 to relieve pain (antihyperalgesia) for months. It is therefore indicated that reducing orthodontic pain via the modulating immune system is recognized as a highly promising research direction.

## 6. Orthodontic Treatment for Diabetes Patients

Type 1 diabetes mellitus (T1DM) is an insulin-dependent syndrome due to autoimmune disease in juvenile [230]. Children with diabetes have higher risks for the development of periodontal disease. T1DM is capable of disrupting enamel and dentine formation, while accelerating tooth eruption [168–170] [231–233], which can influence orthodontic treatment. However, except for controlling blood-glucose before treatment, there is no systemic strategy for patients with T1DM. Besides, compared with type 2 diabetes mellitus (T2DM) that commonly occurs in elderly, pathogenesis of T1DM is characterized by autoimmune destruction of insulin-producing beta cells in the pancreas [234] and accompanied by insulitis and increasing of serum proinflammatory cytokines level [235]. In theory, this immunoregulatory alteration influences orthodontic outcome, whereas there has been no solid evidence to support it, and its exact mechanisms remain unclear. There is research suggested that increased high-density lipoprotein (HDL) in T1DM may be detrimental to endothelial function [236]. It can increase permeability of periodontal capillary and magnify inflammation reaction in orthodontic patients. The above theory is also indirectly supported by that higher expression of MMP 8 and 9 and accelerated tooth movement in diabetes rat model [237]. However, there has been no associated clinical data. Further investigations are expected to illuminate and eliminate adverse effects arising from diabetes-associated immune disorders on OTM.

## 7. Orthodontic Relapse

Existing research has suggested that orthodontic relapse is a common problem affecting the outcome of orthodontic treatment. It is hard to predict which patients are at risk of relapse and the extent of relapse in the long-term [238]. The cellular process of orthodontic relapse is similar to OTM. As teeth move in the alveolar bone, the periodontal ligament and gingivae remodel to the new position. Before they completely adjust, teeth can move along the direction of their original position due to the stretching of supra-alveolar gingival fibers, accompanied by osteoclast distribution shifting [238, 239]. Accordingly, it is unsurprising that NSAIDs is capable of reducing orthodontic distance relapse via regulating immune system in rats. Possible mechanisms comprise downregulating proinflammatory cytokines in PDL tissue, blocking CD4+ T-lymphocyte cells and TH1 cells significant for OTM in spleen, and inhibiting PGE2 to suppress osteoclast activation [5]. In addition, M2 macrophages play a critical role in the cessation of bone resorption and the initiation of tissue repair. Thus, abnormal M1/M2 ratio can lead to continuous bone resorption, and tooth movement even though orthodontic force is removed [16, 240]. In-depth investigations on the immune-alveolar bone interaction in relapse are required to foresee risk for relapse and find a novel method of relapse prevention to replace or assist conventional orthodontic retainer.

## 8. Conclusion

Immune system plays a certain role in OTM in numerous aspects. In this paper, factors correlated with immunomodulation are illustrated, and their possibility of acting as potential targets to improve orthodontic outcome or prevent complications is discussed.

## Data Availability

All data used during the study appear in the submitted article.

## Abbreviations

5-HT: 5-Hydroxytryptamine
*α*-SMA: Alpha-smooth muscle actin
ACD: Allergic contact dermatitis
APCs: Antigen presenting cells
ASIC3: Acid-sensing ion channel 3
BMPs: Bone morphogenetic proteins
BPA: Bisphenol A
BMSCs: Bone marrow stromal cells
C5: Complement component 5
CCL20: Chemotactic cytokines ligand 20
CGRP: Calcitonin gene related peptide
DCs: Dendritic cells
DTH: Delayed-type hypersensitivity
ERR: External root resorption
ESR: External surface resorption
GC: Glucocorticoid
GCF: Gingival crevicular fluid
HDL: High-density lipoprotein
HGF: Human gingival fibroblasts
HIF-1: Hypoxia-inducible factor-1
HSCs: Hematopoietic stem cells
hVDR: Human vitamin D receptor
ICAM-1: Intercellular adhesion molecule 1
IGFs: Insulin-like growth factors
KGF: Keratinocyte growth factors
LCs: Langerhans cells
LPS: Lipopolysaccharide
LTB4: Leukotriene B4
MAPK: Mitogen-activated protein kinase
M-CSF: Macrophage colony-stimulating factor
MHC II: Major histocompatibility complex II molecule
MMP-8: Matrix metalloproteinases 8
NF-*κ*B: Nuclear factor-*κ*B
NGF: Nerve growth factor
NK cells: Natural killer cells
OC: Osteocalcin
OPN: Osteopontin
OTM: Orthodontic tooth movement
PAG: Periaqueductal gray
PAR-2: Protease activated receptor 2
PDL: Periodontal ligament
PDLC: Periodontal ligament cell
PDLSC: Periodontal ligament stem cells
PGE_2_: Prostaglandin E_2_
PMMA: Polymethyl methacrylate
RANKL: Receptor activator of NF-*κ*B ligand
SP: Substance P
T1DM: Type 1 diabetes mellitus
T2DM: Type 2 diabetes mellitus
TGF-*β*: Transforming growth factor-beta
TLR4: Toll-like receptor 4
Tregs: Regulatory T cells
TRPV1: Transient receptor potential cation channel subfamily V member 1
VEGF: Vascular endothelial growth factor.
